# Update of the current knowledge on genetics, evolution, immunopathogenesis, and transmission for coronavirus disease 19 (COVID-19)

**DOI:** 10.7150/ijbs.48812

**Published:** 2020-09-12

**Authors:** Kalthoum Tizaoui, Ines Zidi, Keum Hwa Lee, Ramy Abou Ghayda, Sung Hwi Hong, Han Li, Lee Smith, Ai Koyanagi, Louis Jacob, Andreas Kronbichler, Jae Il Shin

**Affiliations:** 1 Laboratory Microorganismes and Active Biomolecules, Sciences Faculty of Tunis, University Tunis El Manar, Tunis, Tunisia.; 2 Department of Pediatrics, Yonsei University College of Medicine, Seoul, Republic of Korea.; 3 Division of Urology, Brigham and Women's Hospital and Harvard Medical School, Boston, MA, USA.; 4 Department of Global Health and Population, Harvard T.H. Chan School of Public Health, Boston MA, USA.; 5 Yonsei University College of Medicine, Seoul, Republic of Korea.; 6 University of Florida College of Medicine, Gainesville, FL 32610, USA.; 7 The Cambridge Centre for Sport and Exercise Science, Anglia Ruskin University, Cambridge, CB1 1PT, UK.; 8 Research and Development Unit, Parc Sanitari Sant Joan de Déu, CIBERSAM, 08830 Barcelona, Spain.; 9 ICREA, Pg. Lluis Companys 23, 08010 Barcelona, Spain.; 10 Faculty of Medicine, University of Versailles Saint-Quentin-en-Yvelines, 78000 Versailles, France.; 11 Department of Internal Medicine IV (Nephrology and Hypertension), Medical University Innsbruck, Innsbruck 6020, Austria.

**Keywords:** COVID-19, SARS-CoV-2, genetic variation, SARS-CoV-2 genomics, evolution, Immunity, transmission

## Abstract

In December 2019, an acute respiratory disease caused by novel species of coronavirus (SARS-CoV-2), emerged in China and has spread throughout the world. On 11^th^ March 2020, the World Health Organization (WHO) officially declared coronavirus disease 19 (COVID-19) a pandemic, severe coronavirus-mediated human disease. Based on genomic and phylogenetic studies, SARS-CoV-2 might originate from bat coronaviruses and infects humans directly or through intermediate zoonotic hosts. However, the exact origin or the host intermediate remains unknown. Genetically, SARS-CoV-2 is similar to several existing coronaviruses, particularly SARS-CoV, but differs by silent and non-silent mutations. The virus uses different transmission routes and targets cells and tissues with angiotensin-converting enzyme 2 (ACE2) protein, which makes it contagious. COVID-19 shares both the main clinical features and excessive/dysregulated cell responses with the two previous Middle East respiratory syndrome coronavirus (MERS) and severe acute respiratory syndrome coronavirus (SARS) epidemics. In this review, we provide an update of the current knowledge on the COVID-19 pandemic. Gaining a deeper understanding of SARS-CoV-2 structure, transmission routes, and molecular responses, will assist in the prevention and control of COVID-19 outbreaks in the future.

## Introduction

The human community has experienced outbreaks and epidemics of many infectious diseases which are considered as major causes of human morbidity and mortality. Viral pathogens are known to cause outbreaks that have epidemic and pandemic potential. Several factors underlie the emergence of such diseases, including increasing population, increased domestic and global connectivity, social practices, prevalence of immunosuppressive diseases, change in agricultural practices such as mixed farming, and many other related environmental factors 1. Climate changes and reduction in biodiversity may play a role by forcing species to change geographical range and to survive in seminatural habitats that may bring wild animals closer to humans 2. Disturbance of natural ecosystems is reported to increase the transfer of disease from wild species to humans and is suggested to increase occurrences of neglected, forgotten and new human diseases 3. It is well known that pathogen genomics vary considerably, thus, occurring genetic alterations have also been responsible for such outbreaks 4. Although humanity has made meaningful progress in the battle against pathogens, the re-emergence of viral agents remains a great threat and challenge for the global health community 5. About 60% of infectious diseases and 70% of emerging infections of humans are zoonotic in origin, with two-thirds originating in wildlife 6. Over the last decades, several viral diseases had emerged in China, namely, those caused by the severe acute respiratory syndrome- (SARS-) coronavirus (CoV) and the H5N1, H1N1, and H7N9 viruses 7. Recently, near the end of 2019, an outbreak of an ongoing viral pneumonia with unknown etiology emerged in the city of Wuhan, China. The infectious agent of this viral pneumonia was identified as the 7^th^ member of human coronaviruses (2019-nCoV) 8. On 11^th^ February 2020, the World Health Organization (WHO) named the novel viral pneumonia “Coronavirus Disease 2019” (COVID-19), while the International Committee on Taxonomy of Viruses (ICTV) suggested this novel coronavirus will be named “SARS-CoV-2” due to the phylogenetic and taxonomic analysis of this novel coronavirus 9. The SARS-CoV-2 emerged as a highly pathogenic and large-scale epidemic coronavirus compared to the severe acute respiratory syndrome coronavirus (SARS-CoV) in 2002 and Middle East respiratory syndrome coronavirus (MERS-CoV) in 2012. COVID-19 is characterized by a high infectivity during incubation and a time delay between really infected cases and daily observed number of confirmed cases 10. The novel coronavirus is believed to be infectious during its incubation period, which is reported to be 3-7 days, at most 14 days, when no symptoms are shown in patients 11. Environmental factors, particularly weather conditions, are suspected to favor emergence and spread of the outbreak. Both drought and cold, which characterized the 2019 winter in Wuhan 12, provided conducive environmental conditions for virus survival 13,14. During the cold winter, air-dried virus particles are a dangerous form of virus, which survive for a long period of time in airflow 15. In addition, cold conditions damper humans' innate immunity by reducing blood supply and thus decreasing the provision of immune cells to the nasal mucosa. Low humidity can reduce the capacity of cilia cells in the airway to remove virus particles and secrete mucus as well as repair airway cells 16.

Many COVID-19 patients were potentially exposed to wildlife animals at the Huanan seafood whole sale market, Wuhan, China, where poultry, snake, bats, and other farm animals were also sold 17. In such wildlife trade markets, animals known to transmit coronaviruses are crammed together under fetid conditions. Thus, humans might become unfortunate hosts for SARS-CoV-2 as a result of some inappropriate interactions with wildlife. Accumulated evidence suggests bats as the origin of the majority of emerging coronaviruses 18, 19,20,21. Evolution and recombination of these different strains of bat viruses might lead to the creation of various SARS-CoVs capable of cross-species transmission and ultimate infection of human beings 22, 23, 24, 25. Considering that the earliest COVID-19 patient reported no exposure at the seafood market, the suspected source of first virus infections 17, it becomes vital to identify the intermediate SARS-CoV-2 host to block interspecies transmission. Currently, the research on SARS-CoV-2 is in its primary stages. Based on current published evidence, this review summarizes SARS-CoV-2 origin, genetics, genomics, transmission routes, immune system responses and immunopathogenesis of COVID-19.

## Origin of COVID- 19

Recently (at the time of writing this paper), the worldwide scientific community released full genomic sequences and several independent research groups have identified SARS-CoV-2 as a β-coronavirus. It is an enveloped virus containing a non-segmented, positive-sense RNA molecule 8,26,27. In assigning the SARS-COV-2 to a taxonomic group, phylogenetic analysis of the complete viral genome revealed that the virus was most closely related (89.1% nucleotide similarity) to a group of SARS-like β-coronaviruses (β-CoVs) previously found in bats in China 28. Sequencing and evolutionary analyses show that the bat was suspected as a natural reservoir host of the virus 19-21. Indeed, the SARS-CoV-2 genome sequence is identical to those of bat CoVs, particularly to the RaTG13, with similarities from 96.2% to 99.9% 22,26,29-31. SARS-CoV-2 might be transmitted from bats via unknown intermediate hosts to humans. Bats are the natural reservoir of a wide variety of CoVs, including SARS-CoV-like and MERS-CoV-like viruses 32-34. The bat SARS-like coronavirus sequence is genetically more similar to the 2019-nCoV than other bat SARS-like coronavirus sequences, but is also more distant from sequences isolated in SARS and MERS coronaviruses 35. A possible explanation is a past history of recombination in the β-coronavirus group 18. Coronaviruses are characterized by a high rate of recombination, which may play a role in viral evolution and interspecies infections 36,23.

**Figure 1:**
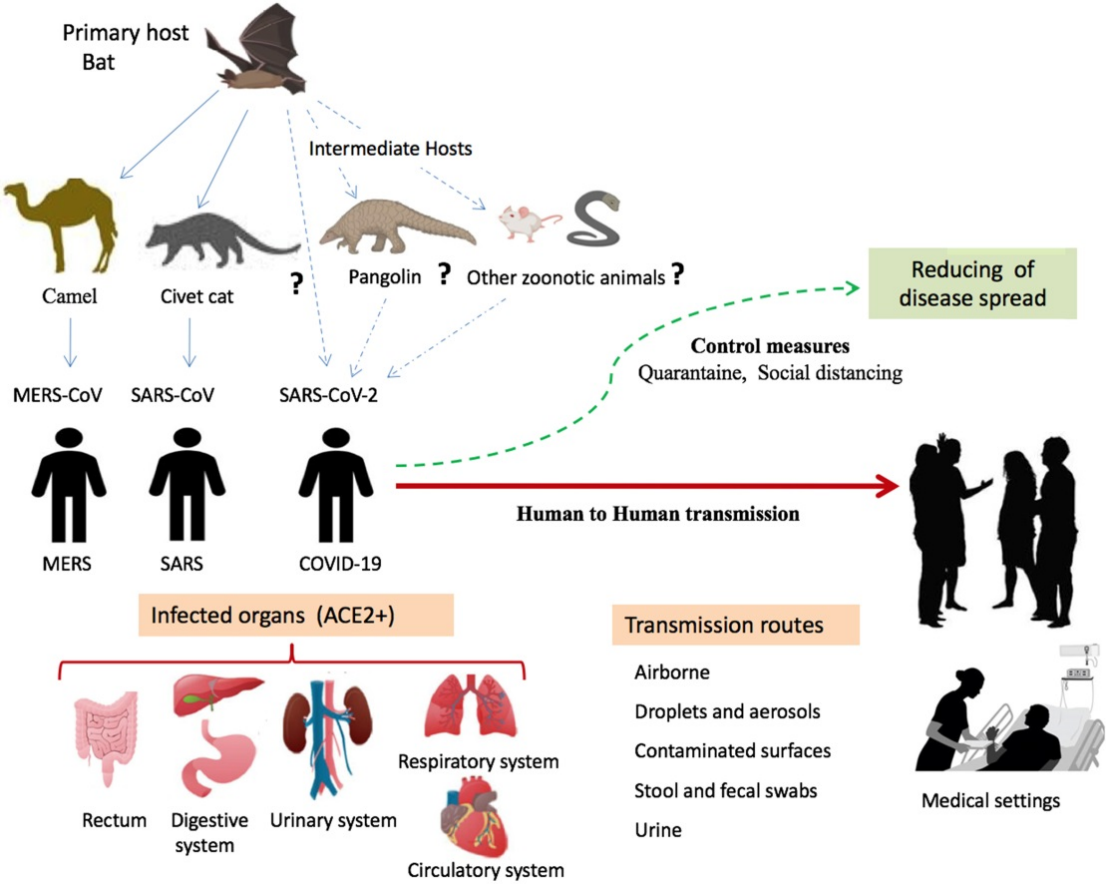
** Origin of SARS-CoV-2 and potential transmission routes to humans.** SARS-CoV-2 transmitted to humans from bats directly or by intermediate hosts such as rats, pangolins, snakes, and rats. The virus is spread among humans by different routes including droplets, aerosols, direct contact, and other potential routes such as urine, stool, and fecal swabs. Human organs expressingthe ACE2 receptor are targets for viral infection. Abbreviation: ACE2+, organs having ACE2 receptor.

Given that bats were not available for sale in the seafood market in Wuhan and similar residues of virus receptor were observed in many species 24, alternative intermediate hosts, such as turtles, pangolin and snakes were proposed 23,24. Usually, SARS-CoV and MERS-CoV infect intermediate hosts, such as civets or camels, before leaping to humans 37. This fact indicates that SARS-CoV-2 was probably transmitted to humans from animals other than bats. Some studies suggested pangolin-CoV is the closest relative of SARS-CoV-2 38,39. On 24^th^ October 2019, Liu and colleagues detected the existence of a SARS-CoV-like CoV in lung samples collected from two dead Malayan pangolins 38. This discovery was made just prior to the COVID-19 outbreak. Interestingly, Pangolin-CoV was composed of a 91.02 % identical genome to SARS-CoV-2 39. Notably, Paraskevis et al. 20 reported that the new coronavirus provides a new lineage for almost half of its genome, with no close genetic relationships to other viruses within the subgenus of sarbecovirus, rejecting the hypothesis of SARS-CoV-2 emergence as a result of a recent recombination event. Taken together, genomic and phylogenetic results indicate that the SARS-CoV-2 basal origin is still under debate. **Figure 1** summarizes recent findings on the origin of SARS-CoV-2 and potential transmission routes.

## Immunopathogenesis

Fever, cough, myalgia, or fatigue with abnormal lung scan findings are features of SARS-CoV-2 infection. The less common symptoms are sputum production, headache, hemoptysis and diarrhea 17,40,41. Few patients develop acute respiratory distress syndrome (ARDS), respiratory failure, multiple organ failure and even death 17. The elderly and those with underlying disorders (i.e. hypertension, chronic obstructive pulmonary disease, diabetes, cardiovascular disease), are more likely to develop ARDS, cytokine storm, septic shock, metabolic acidosis, and coagulation dysfunction 11,17,42,43. Several abnormalities have been observed including cellular immune deficiency, coagulation activation, myocardial injury, hepatic and kidney injury, and secondary bacterial infection 43.Of note, scientists reported that SARS-COV-2 causes an inflammatory response in the lower airway, which may lead to lung injury 44,45. Viral particles invade the respiratory mucosa, triggering immune responses and a “cytokine storm” closely related to the critical condition of COVID-19 patients 46. COVID-19 symptoms showed differences in viral tropism compared to SARS-CoV 11,42,45, MERS-CoV 46 and influenza virus 47. Innate immunity serves to slow viral infection before the adaptive immune response. IFN-γ secretion constitutes the first anti-viral defense barrier. In the case of SARS-CoV-2, IFN-γ secretion might be dampened by viral replication and subsequent inhibition of the adaptive immune response. Moreover, viral replication triggers hyper inflammatory conditions, leading to a high production of pro-inflammatory cytokines and chemokines, particularly by neutrophils and monocytes/macrophages 48-50. Several plasma cytokines and chemokines were increased in COVID-19 patients, including IL-1, IL-2, IL-4, IL-7, IL-6, IL-10, IL-12, IL-13, IL-17, G-CSF, M-CSF, IP-10, MCP-1, MIP-1α, HGF, IFN-γ and TNF-α 17,24. Normally, produced cytokines and chemokines play an anti-viral defense role through the recruitment of immune cells. However, in critical cases of COVID-19 patients, a “cytokine storm” is reported and exacerbates immune reactions, initiating viral sepsis and ARDS. These exacerbations lead to death in some cases of elderly and those with underlying disorders 17.

**Figure 2:**
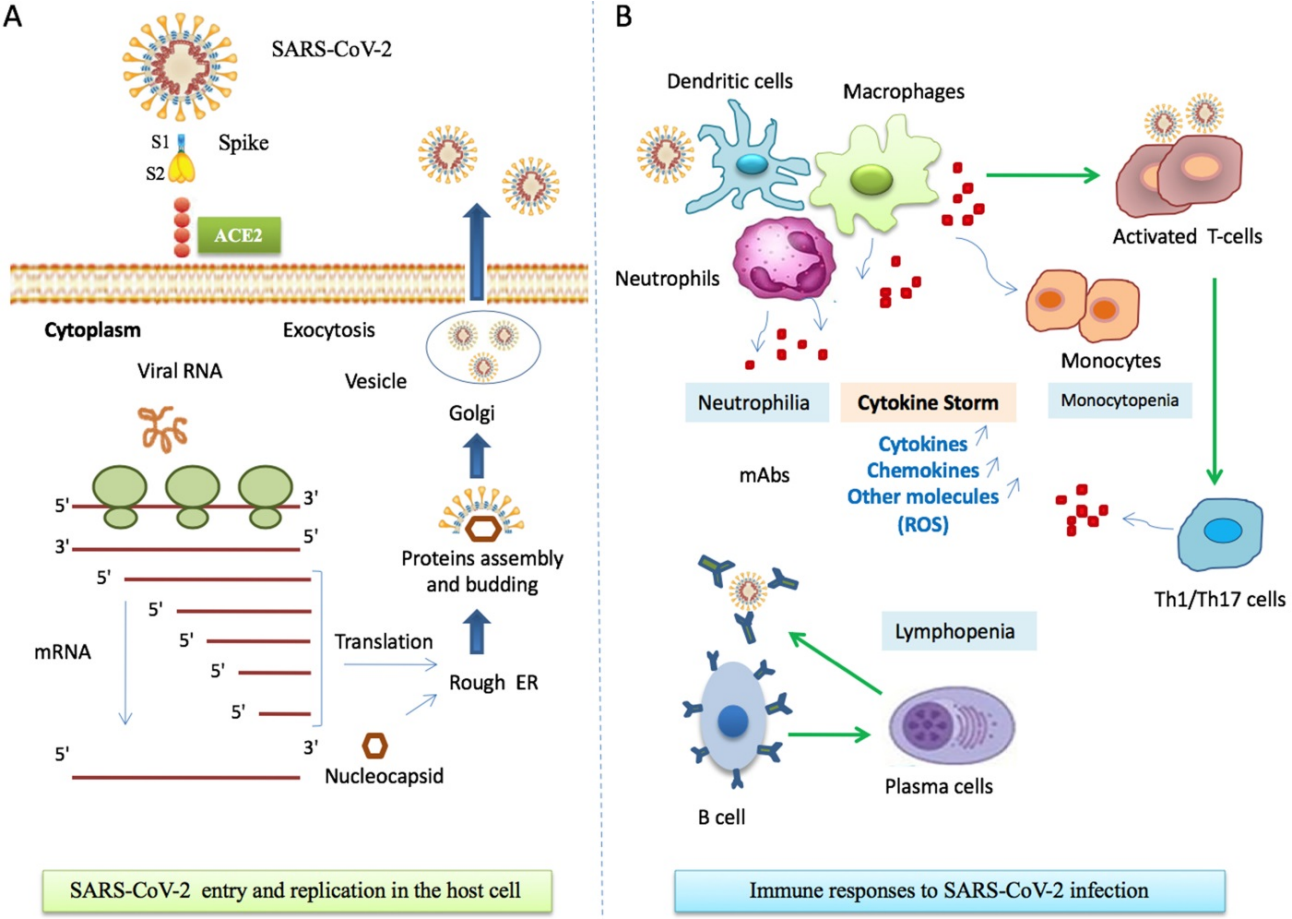
** Immune responses during SARS-CoV-2 infection. A.** SARS-CoV-2 entry to host cell. Spike proteins on the surface of the coronavirus bind to angiotensin-converting enzyme 2 (ACE2) receptors on the surface of the target cell. Coronavirus genome replication and transcription takes place in the cytoplasmand involves coordinated processes of both continuous and discontinuous RNA synthesis. Assembly of viral proteins occurred in rough RE. Finally, the virus is released through exocytosis by Golgi. Abbreviations: S1, Spike 1; S2, Spike 2; ACE2, angiotensin-converting enzyme 2, RE, reticulum endoplasmic. **B.** COVID-19 immuno-pathogenesis. Infected cells present CoV antigens to T cells. This process leads to T cell activation and differentiation, including the production of cytokines associated with the different T cell subsets (Th1/Th17). Cytokines recruit lymphocytes and leukocytes to the site of infection. The adaptive immunity is involved through a subset of differentiated Tcells and activation of B and plasma cells that produce monoclonal antibodies. Activation of immune cells results in excessive productionof chemokines and other cytokines that induce a pro-inflammatory response and attract cells, such as neutrophils and macrophages, to sites of infection. In turn, these cells release injury molecules, such as matrix metalloproteinases and ROS. The “cytokine storm” represents the secretion of large quantities of immune mediators leading to more severe conditions. Regulated immune responses are crucial to clear the infection. Abbreviations: mAbs, Monoclonal Antibodies; Th, T helper; ROS, Reactive Oxygen Species.

Most patients have normal or decreased white blood cell counts, and lymphocytopenia 51. However, in severe patients, the neutrophil count, D-dimer, blood urea and creatinine levels are significantly enhanced, while the lymphocyte counts are clearly reduced. The innate immune system recognizes viral 'molecular patterns' (such as double-stranded RNA) 52 and the adaptive immune systems kills virus-infected cells by means of T and B cells that produce pathogen-specific antibodies 53. Regulated immune response inhibits virus replication, promotes virus clearance, induces tissue repair, and triggers a prolonged adaptive immune response against the viruses 54. **Figure 2** describes the mechanisms of host cell infection by SARS-CoV-2 and gives a simplified schema of the immunopathogenesis of COVID-19.

## SARS-COV-2 genomics and variation

Coronaviruses are single-stranded RNA viruses that belong to the order Nidovirales, family Coronaviridae, and subfamily Coronavirinae 54. The family Coronavirinae is divided into four genera: α, β, γ, and δ. There are seven human coronaviruses: 229E (α-CoV), NL63 (α-CoV), OC43 (β- CoV), HKU1 (β-CoV), MERS-CoV (β-CoV), SARS-CoV (β-CoV), and SARS-CoV-2 (β-CoV) 55. The viral genome of SARS-COV-2 is composed of a positive-stranded RNA, and its structures vary considerably 8. The complete genome of one strain of SARS-CoV-2, isolated from a COVID-19 pneumonia patient working in the Wuhan seafood market, is 29.9 kilo-bases (kb) in size with 29891 nucleotides 56, encoding 9860 amino acids 57, while SARS and MERS-CoVs have RNA genomes of 27.9 kb and 30.1 kb, respectively 58. Similar to other β-CoVs, the SARS-CoV-2 genome contains two flanking untranslated regions (UTRs) and a variable number (8-13) of open reading frames (ORFs) 59. Two-thirds of viral RNA, mainly located in the first ORF (ORF1a/b) translates two polyproteins, pp1a and pp1ab, and encodes 16 non-structural proteins (NSP). The remaining ORFs encode accessory and structural proteins 37. The 2019-nCoV genome is arranged in the order of 5′-replicase (ORF1 /ab)-structural proteins [Spike (S)-Envelope (E)- Membrane (M)-Nucleocapsid (N)]-3′. The 5′- and 3′ -UTR sequences of 2019-nCoV, 265 and 358 nucleotides respectively in length, are similar to those of other β-CoVs, with 83.6% shared nucleotide identity. There are no remarkable differences between the ORFs and NSPS of 2019-nCoV with those of SARS-CoV 57. The major distinction between 2019-nCoV and SARS-CoV is in spike S1 and ORF8, which were previously shown to be recombination hot spots 57. In correlation with previous knowledge of CoVs genomics 60, the AT% was higher than GC% in SARS-CoV-2 49. In all of its structural genes, SARS-CoV-2 prefers pyrimidine rich codons to purines, and most high-frequency codons ended with A or T. The low-frequency codons ended with G or C 61. This is in agreement with previous studies of CoVs 62. SARS-CoV-2 structural proteins showed 5-20 lower ENc values compared to SARS, bat SARS and MERS CoVs. This implies higher codon bias and higher gene expression efficiency of SARS-CoV-2 structural proteins 61. By meta-transcriptomic sequencing, another study showed that SARS-CoV-2 exhibits some genomic and phylogenetic similarity to SARS-CoV, particularly in the S-glycoprotein gene and receptor-binding domain (RBD) 56. Most genome-encoded proteins of SARS-CoV-2 are similar to SARS-CoVs 56. Mutations in NSP2 and NSP3 play a role in the infectious capability and differentiation mechanism of SARS-CoV-2 63.

Phylogenetic and genomic analyses from distinct countries suggest the newly-emerged SARS-CoV-2 strains are closely related but are distinguished by both synonymous and non-synonymous mutations in different genomic locations 18,19,21,22,29,36,64-70. Genome sequences of 2019-nCoV sampled from early cases were almost genetically identical 17,21, suggesting a very recent emergence of this virus in humans. The estimated genetic diversity of five newly sampled 2019-nCoV genomes was 0.000094 substitutions per site with an estimated evolutionary rate of 0.0038 substitutions per site per year 65. Accordingly, the newly identified 2019-nCoV sequences originated from the same isolate about 2 years ago 64. Evolutionary selection in the human hosts acts on SARS-COV-2 genomes, sometimes with parallel evolution events. Tang et al. 66 conducted a population genetic analysis of 103 SARS-CoV-2 genomes and classified two prevalent evolving types of SARS-CoV-2: L type (~ 70%) and S type (~ 30%). Likewise, the complete genome analysis for the first cases of COVID-19 in Chile detected at least two different viral variants 67. The new coronavirus could face selective pressures such as diversity in hosts, countries, weather, and other conditions. The phylogeographic patterns are potentially affected by distinctive migratory histories, founder events, and sample sizes 64. This information contributes to monitoring the spread of the infection and the surveillance for eventual recombination or genome mutations. Population genetics-phylogenetics approach indicated that most sites in the viral ORFs evolved under strong to moderate purifying selection. Particularly, a non-negligible proportion of ORF8 codons had evolved under very weak purifying selection or close to selective neutrality 30. Positive selection was also detected in the receptor-binding motif (RBM) of the spike protein but most likely resulted from a recombination event that involved the BatCoV RaTG13 sequence 30. The divergence of SARS-CoV-2 from BatCoV RaTG13 was accompanied by limited episodes of positive selection, suggesting the common ancestor of the two viruses was poised for human infection 30.

Homologous recombination contributes to the 2019‐nCoV cross‐species transmission 23. Homologous recombination is an important evolutionary force that occurs in many viruses, including Dengue virus 71, human immunodeficiency virus 72, hepatitis B virus 73, and hepatitis C virus 74. A previous study suggested that recombination of SARS-CoV in the spike glycoprotein genes might have mediated the initial cross-species transmission event from bats to other mammals 25. It is critical to determine the animal reservoir of the 2019‐nCoV to understand the molecular mechanism of its cross‐species spread. Findings shed a cautiously optimistic light on the possibility of finding effective treatment for this novel coronavirus, starting from already existing anti‐β-coronaviridae compounds 29.

## The process of infection in human

In the past two decades, the 2019-nCoV is the third coronavirus to emerge in the human population after the SARS-CoV outbreak in 2002 75 and the MERS-CoV outbreak in 2012 76. Full-genome sequence analysis of 2019-nCoV is different from both MERS-CoV and SARS-CoV that infect humans 8, but all are highly pathogenic zoonotic pathogens. Based on their phylogenetic relationships and genomic structures, human β-coronaviruses (SARS-CoV-2, SARS-CoV, and MERS-CoV) have many similarities but also have differences in their genomic and phenotypic structures that influence their pathogenesis. To date, no therapeutics or vaccines are approved against any human-infecting coronaviruses. Epidemiological investigations suggest SARS-CoV-2 is highly transmissible in humans 77, especially in the elderly and people with underlying diseases 78. The rapidly increasing number of cases and evidence of human-to-human transmission suggest the virus was more contagious than SARS-CoV and MERS-CoV 79,80. WHO estimates, the basic reproduction number (R0), as 1.4 to 2.5 less than SARS (2 to 5); but, this number can grow if the pandemic is not controlled by applying quarantine and isolation strategies 21. The high affinity between ACE2 and 2019-nCoV spike protein also suggests the population with a higher expression of ACE2 might be more susceptible to 2019-nCoV 81. The SARS-CoV first emerged in China in 2002 and then spread to 37 countries/territories in 2003 and caused a travel-related global outbreak with a 9.6% mortality rate 82. There is evidence that SARS-CoV originated in bats in China and reached humans after jumping from an intermediate host, the civet (Pagumalarvata) 83. MERS-CoV, discovered in 2012 in the Middle East 84, is endemic in dromedary camels, from which it can be transmitted to humans 20. The MERS epidemic is ongoing, and as of December 2019, 2468 cases have been reported 85. Less virulent coronavirus species cause common colds in humans with relatively mild symptoms 86. Some coronaviruses are strictly host-specific, while others can be found in a range of hosts 87.

Genetically, SARS-CoV-2 was less similar to SARS-CoV (about 79%) and MERS-CoV (about 50%) 88,89. The arrangement of nucleocapsid protein (N), envelope protein (E), and membrane protein (M) among β-coronaviruses are different. Both SARS-CoV-2 and SARS-CoV bind to human ACE2 90 and use transmembrane protease serine 2 (TMPRSS2) to complete cell entry and infection 89,91. The SARS-COV-2 spike protein has a furin cleavage site in the S1/S2 junction, different from SARS-CoV and other closely related bat viruses 92. This has implications for viral entry routes. The TMPRSS2 also contributes to the S-protein priming of 2019-nCoV, indicating that the TMPRSS2 inhibitor might constitute a treatment option 89. There are many similarities between SARS-CoV-2 and the original SARS-CoV 93, and their S proteins share 76.47% identity 93,94. A total of 53 unique S proteins were selected and their structures modeled according to different subtypes of CoVs, including 2019-nCoV (WH-Human_1), 3 SARS strains, 2 β-CoV strains, and 47 strains from other CoVs. S proteins of 2019-nCoV and SARS strains share high structural similarity with a root-mean-square deviation of 1.21 Å 94. Wan et al. 94 reported that residue 394 (glutamine) in the SARS-CoV-2 RBD, corresponding to residue 479 in SARS-CoV 95, can be recognized by the critical lysine 31 on the human ACE2 receptor. A highly similar epitope was identified computationally between the 2019-nCoV and SARS-CoV on the binding site of the S proteins to the human ACE2 receptor 55. Further analysis even suggested SARS-CoV-2 recognizes human ACE2 more efficiently than SARS-CoV, increasing the ability of SARS-CoV-2 to transmit from person to person 94.

## Potential transmission routes of SARS-CoV-2

As an emerging acute respiratory infectious disease, COVID-19 primarily spreads through the respiratory tract by droplets, respiratory secretions, saliva, and direct contact for a low infective dose 42,96. The common transmission routes of novel coronavirus include direct transmission (cough, sneeze, droplet inhalation transmission) and contact transmission with oral, nasal, and eye mucous membranes 97. Since 2019-nCoV can be passed directly from person to person by respiratory droplets, emerging evidence suggests it may also be transmitted through contact and fomites 98. The fact that SARS-CoV-2 can infect the human gut epithelium has important implications for fecal-oral transmission and containment of viral spread 99. The ACE2 receptor is abundantly present throughout the respiratory tract, as well as in the epithelial cells of salivary gland ducts that have been demonstrated to be early targets of SARS-CoV 100. Both SARS-CoV and SARS-Cov-2 may have much faster replication rates than other coronaviruses infect humans 94 . The 2019-nCoV can produce aerosols, droplets, or particulate matter with high viral loads that increase the viability time of the virus in various environments 100. After reaching the recipients, the fast replication viruses have a higher chance of successful infection 100. Of note, a report of one case of 2019-nCoV infection in Germany indicates that transmission of the virus may also occur through contact with asymptomatic patients 97. In fact, the asymptomatic incubation period for individuals infected with 2019-nCoV has been reported to be ~1-14 days. Thus, those without symptoms can spread the virus 101.

On 10^th^ February 2020, SARS-CoV-2 was isolated from fecal swabs from a severe pneumonia patient in China 102, indicating the possibility of multiple routes of transmission. In addition, a recent pilot experiment showed that 4 out of 62 stool specimens tested positive for 2019-nCoV, and another four patients in a separate cohort who tested positive from rectal swabs also had 2019-nCoV detected in the gastrointestinal tract, saliva, or urine 30. Thus, in addition to the respiratory droplets and direct contact, fecal-oral transmission might also be a route of transmission for 2019-nCoV 40. Remarkably, the ACE2 protein presents abundantly on enterocytes in the small intestine 103, which may contribute to this route of infection and disease manifestations. However, the aerosol transmission route and the fecal-oral transmission routes still need to be further studied.

Analysis of conjunctival samples from confirmed and suspected cases of 2019-nCoV suggests eye exposure may provide an effective way for the virus to enter the body 104, and that ocular surfaces may be a potential target for SARS-CoV-2 invasion 98. However, other studies do not fully support this assumption 105. ACE2 is mainly expressed in posterior tissues of the eye, such as the retina and the retinal pigment epithelium, not in the human conjunctival and corneal epithelium 106. Furthermore, tears are constantly renewed by the lacrimal drainage system. Therefore, the virus enters the tears through droplets, which may pass through the naso-lacrimal ducts and then into the respiratory tract 88.

Asymptomatic patients can spread SARS-CoV-2 with high efficiency in noninfectious disease settings such as otolaryngology, which is a high-risk specialty as it closely contacts the upper respiratory tract mucous, secretions, droplets and aerosols during procedures and surgery 107, 108. Otolaryngologists have been infected with COVID-19 at higher rates than other specialties 107. Of note, pathogens can be transmitted in dental settings through inhalation of airborne microorganisms that can remain suspended in the air for long periods 109. Direct contact with blood, oral fluids, contact of conjunctival, nasal, or oral mucosa with droplets and aerosols containing microorganisms generated from an infected individual 110, and indirect contact with contaminated instruments and/or environmental surfaces 111, can rapidly spread viral pathogens. Dental studies show that many dental procedures and contaminated dental instruments or environmental surfaces provide possible routes to the spread of viruses 112,113. Human coronaviruses such as SARS-CoV, MERS-CoV, or endemic human coronaviruses (HCoV) can persist on surfaces like metal, glass, or plastic for up to a couple of days 114, making contaminated surfaces in healthcare settings a potential source of coronavirus transmission.

## Key viral factors

The coronaviral genome encodes four structural proteins, namely, S protein, N protein, M2 protein, and E protein 54,115. The N protein interacts with the viral RNA to form the ribo-nucleoprotein 116. The E protein conducts ion channel actions and contributes to virions assembly 117. Coronavirus entry to host cells is a multi-step process involving several distinct domains in the surface glycoprotein spike (S). The S protein, a trimeric class I fusion protein, exists in a metastable prefusion conformation that undergoes a substantial structural rearrangement to fuse the viral membrane with the host cell membrane 118,119. The S protein contains the RBD and mediates virus attachment to the cell surface, receptor engagement, protease processing, and membrane fusion, facilitating viral entry into host cells 120-124. The virion S-glycoprotein on the coronavirus surface can attach to the ACE2 receptor on the surface of human cells 125. The S proteins mutate and gain capability to recognize host receptors across species 118,126. For many CoVs, spike is cleaved at the boundary between the S1 and S2 subunits 121. For SARS-CoV, the cleavage of the trimer S protein is triggered by the cell surface-associated TMPRSS2 127 and cathepsin 128. To engage a host cell receptor, the RBD of S1 undergoes hinge-like conformational movements that transiently hide or expose the determinants of receptor binding 129. The RBD for SARS-CoV-2 has residues and motifs found in all three clades but forms a distinct clade 94. Receptor binding destabilizes the pre-fusion trimer, resulting in shedding of the S1 subunit and transition of the S2 subunit to a stable post-fusion conformation 130.

After membrane fusion, the RNA viral genome is released into the cytoplasm, and the uncoated RNA translates two polyproteins, pp1a and pp1ab 130, which encode non-structural proteins and form a replication-transcription complex (RTC) in double-membrane vesicles. RTCs replicate and synthesize a nested set of subgenomic RNAs 131, which encode accessory proteins and structural proteins. Mediated by the endoplasmic reticulum (ER) and the Golgi, newly formed genomic RNA, nucleocapsid proteins, and envelope glycoproteins assemble and form viral particle buds 131.

## SARS-CoV-2 and ACE2

The ACE2 protein is enriched in the enterocytes in the small intestine and the renal tubules, as well as in the lung alveolar epithelial cells, the heart cells, the arterial smooth muscle cells, and the gastrointestinal system 133,134. This protein is best known for cleaving several peptides within the renin-angiotensin system and other substrates 135. ACE2 protein is rare in circulation, but widely expressed in organs, such as kidneys, the gastrointestinal tract, and at relatively lower levels, the lungs 136. Recently, based on scRNA-seq datasets, Zou et al. 105 constructed a 2019-nCoV infection-related risk map of different organs including nasal mucosa, respiratory tract, bronchus, and lung. They found that pulmonary AT2 cells and respiratory epithelial cells exhibited high ACE2 expression. Similarly, Zhao et al. 81 demonstrated that 83% of ACE2-expressing cells were alveolar epithelial type II cells (AECII), suggesting these cells can serve as a reservoir for viral invasion. In addition, gene ontology enrichment analysis showed ACE2-expressing AECII have high levels of multiple viral process-related genes, including regulatory genes for viral processes, viral life cycle, viral assembly, and viral genome replication 81, this implies that the ACE2-expressing AECII facilitates coronaviral replication in the lung. Taken together, these data suggest that the respiratory tract should be considered as a vulnerable target to SARS-CoV-2 infection.

For the human heart, more than 7.5% of myocardial cells demonstrate positive ACE2 expression, implying that the heart could be at high risk of 2019-nCoV infection, especially in the presence of viremia 105. Moreover, myocardial infarction may increase ACE2 expression in heart, thereby suggesting that ACE2 plays an important role in cardiac injury 137. Interestingly, the digestive system, including the esophagus, stomach, ileum, and liver, showed extremely high ACE2 expression in epithelial cells 81,105,138,139. In addition, proximal tubule cells in the kidney and bladder urothelial cells express ACE2, making kidney and bladder a sat risk for infection 103,105,140. Taken together, ACE2 tissue distribution in different organs could explain the multi-organ dysfunction and the non-respiratory symptoms observed in some 2019-nCoV pneumonia patients and may help to explain the increased human-to-human transmissibility of this virus 94. The ACE2 extracellular domain has been demonstrated as a receptor for the S protein of SARS-CoV 141, and recently, for the SARS-CoV-2 26. Therefore, accumulated evidence suggests that, similar to SARS-CoV, SARS-CoV-2 uses ACE2 as its host receptor 79,94,105,142 but with higher affinity than does SARS-CoV 79,94,105,143. The similarity with SARS-CoV is critical because ACE2 is a functional SARS-CoV receptor *in vitro* 144 and *in vivo* 145, and this may have implications in therapeutics. Of note, the SARS-CoV-2 does not use other coronavirus receptors such as aminopeptidaseN and dipeptidylpeptidase 4 31.

During infection, the cleavage of the spike protein required for cell-to-cell fusion depends on both cell type and virus strain 146. Spike protein is proteolytically cleaved by the cellular enzyme furin into S1 and S2 subunits during intracellular processing 123,147. The second cleavage S29 exposes the fusion peptide and is thus necessary for viral entry 108, whereas S1 contains the RBD, which directly binds to the peptidase domain (PD) of ACE2 34. The ectodomain of the SARS-CoV-2 S protein binds to the PD of ACE2 with a dissociation constant (Kd) of~15nM 126. The RBD is recognized by the extracellular peptidase domain of ACE2 mainly through polar residues 148. SARS-CoV-2 and SARS-CoV RBDs show high similarities 94. However, a number of sequence variations and conformational deviations were found 148. A single N501T mutation (corresponding to the S487T mutation in SARS-CoV) may significantly enhance the binding affinity between the 2019-nCoV RBD and human ACE2 94. Of note, for SARS-CoV pathogenesis, ACE2 is not only the entry receptor of the virus but also has a protective role during acute lung injury in a mouse model 145. By binding ACE2, SARS-CoV leads to the downregulation of ACE2 expression and might therefore negate the protective effect of ACE2 145,149. Human and non-human primates share the identity sequences in the regions and residues, implying that ACE2 from non-human primates may recognize 2019-nCoV and mediate its infection 42. As a result, non-human primates may be susceptible to 2019-nCoV and serve as animal models for antiviral research or intermediate hosts for cross-species transmission.

Analysis of coding-region variants using the GTEx database in ACE2 and of the expression quantitative trait loci (eQTL) variants, which may affect the expression of ACE2, support the existence of ACE2 mutants resistant to S-protein binding of coronavirus in different populations 150. East Asian populations have much higher AFs in eQTLvariants associated with higher ACE2 expression in tissues, suggesting different susceptibility or response to SARS-CoV-2 compared to other populations under similar conditions 150. A recent single-cell RNA-sequencing (RNA-seq) analysis indicated that Asian males may have higher expression of ACE2 78. The genetic basis of ACE2 expression and function in different populations is still largely unknown. Increasing understanding of potential functional variants in ACE2 among populations requires further epidemiological investigations of SARS-CoV-2.

## Comparison of SARS-CoV-2 sequences with other coronaviruses

**Figure 3:**
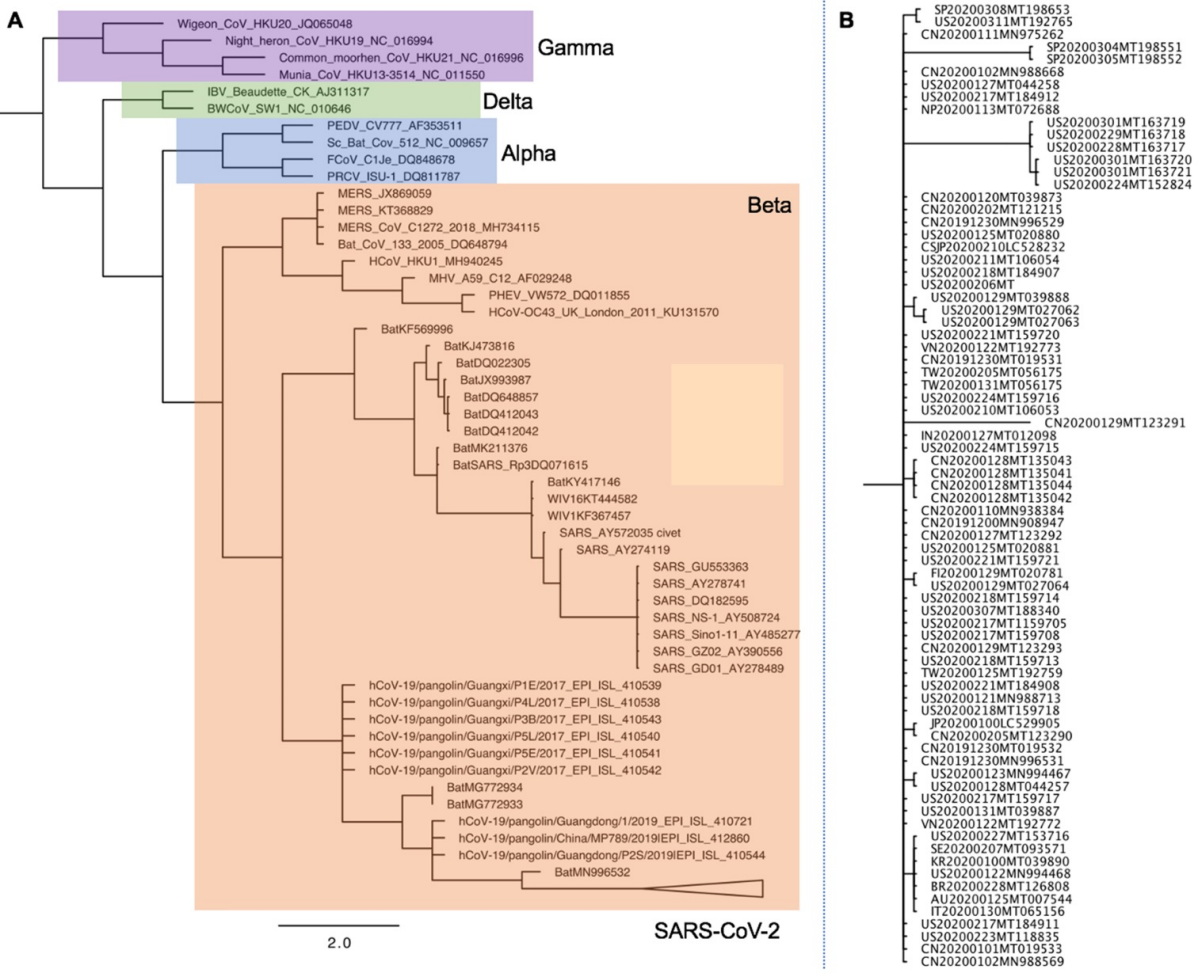
** Phylogenetic analysis of full-length genomes of SARS-CoV-2 and representative sequences of genera Alpha, Beta, Delta-, and Gammacoronaviruses. A.** Phylogenetic analysis of SARS-CoV-2 with Alpha-, Beta-, Delta- and Gammacoronaviruses full-genomes. Colored boxes denote genera. Purple: Gammacoronavirus. Green: Deltacoronavirus. Blue: Alphacoronavirus. Orange: Betacoronavirus. SARS-CoV-2 strains are collapsed in one branch under the Betacoronaviruses. Tree data were collated from updated sources published until June 4^th^, 2020 26,151,152. Abbreviations: CoV, coronavirus; MHV murine hepatitis virus; PHEV porcine hemagglutinating encephalomyelitis virus; IBV infectious bronchitis virus; MERS Middle East respiratory syndrome; SARS severe acute respiratory syndrome. **B.** Phylogenetic analysis of SARS-CoV-2 full-genomes. The collapsed SARS-CoV-2 branch from **A** is shown in full.

Animals have a critical role in COVID-19 outbreak onset and evolution and may act as the virus reservoir. The exact origin of SARS-CoV-2 remains unknown; therefore, phylogenetic analyses are established to find the animal virus reservoir. A phylogenetic analysis comparing the genomes of SARS-CoV-2 with other coronaviruses is shown in **Figure 3** 26,151,152. Nucleocapsid (NC) protein, which is highly conserved and immunogenic, is commonly targeted in studies aimed at developing alternative diagnostic tools 153. Multiple alignments of the whole protein sequences highlight a very high homology between the NC sequence of SARS-CoV-2 and bat RaTG13 CoV 154. Tilocca et al showed that some epitopes are shared among a wider range of coronaviruses, while other epitope sequences are more conserved among the most related specimens 154. Based on the high-homology between the Spike protein epitopes of taxonomically-related coronaviruses, Tilocca et al. hypothesized that past contact with infected dogs shield humans against the circulating SARS-CoV-2 155. By using the Immune Epitope Database and Analysis Resource (IEDB), Grifoni et al. found that SARS-CoV has high sequence similarity to SARS-CoV-2, and is the best characterized coronavirus in terms of epitope responses. Multiple specific regions with high homology to the SARS-CoV in addition to a priori potential B and T cell epitopes for SARS-CoV-2 have been 156. Authors suggest these regions are promising targets for immune recognition of SARS-CoV-2 and can facilitate effective vaccine design against this virus 156.

Compared with other CoV species, bovine CoV is the genetically closest counterpart to human coronaviruses 157,158. A high similarity was observed between bovine CoV, canine respiratory coronavirus (CRCoV) and human coronavirus OC43 (HCoV-OC43) 159. More investigations to assess the transmission rate of the bovine and canine respiratory coronaviruses to humans are needed. Homology modelling showed that 2019-nCoV had a similar RBD structure to that of SARS-CoV, despite amino acid variation at some key residues 26. Lu et al. showed that 2019-nCoV was related (with 88% identity) to two bat-derived severe acute respiratory syndrome (SARS)-like coronaviruses, bat-SL-CoVZC45 and bat-SL-CoVZXC21 and more distant from SARS-CoV (about 79%) and MERS-CoV (about 50%). However, they also revealed that S gene of 2019-nCoV had the lowest sequence identity with bat-SL-CoVZC45 and bat-SLCoVZXC21, at only around 75% 26. Zhou et al. demonstrated that the novel virus has 96.2% similarity to a bat SARS-related Coronavirus (SARSr-CoV; RaTG13 (MN996532.1) 31. Proteins from SARS and SARS-CoV-2 were treated as homologous: identity value > 65%, query coverage, >95% 160. SARS-CoV-2 has been found to be more distant in relation to SARS-CoV (79%) and MERS-CoV (50%) 161. Anand et al. found that all sequences showed ~99.98% similarity in the nucleotide sequences, implying a relationship between the currently circulating viruses and suggesting a recent shift to humans 161.

Comparison of the novel coronavirus Wuhan-Hu-1 sequence with that of the closely related human SARS-CoV S strain Tor2 sequence, revealed 76% homology 162. The S1 RBD was less conserved (64% identity) than the S2 fusion domain (90% identity). The identity between SARS-CoV-2 and SARS-CoV at the S protein amino acid level was 76%, and phylogenetic analyses grouped SARS-CoV-2 in the lineage B of the Betacoronavirus genus, closely related to SARS-CoV, as well as to other CoVs originating in bats 162. The relatively high degree of sequence identity for the RBD is consistent with the view that SARS-CoV-2, like SARS-CoV, may use ACE2 as its host cell receptor 162. The amino acid homology of the modeled S proteins in comparison to the template SARS-CoV S was ~ 71% for all the Bat-CoV S, with the exception of the LYRa3 S, which shares a homology of 84.7% with the template S. Overall, all the modeled S proteins shared a similar folding pattern in comparison to SARS-CoV S and both S1 and S2 domains showed a uniform organization 162. The S protein amino acid identity among the Bat-CoV ranged between 75.3% and 96.7%, with LYRa3 and RaTG13 S proteins having the lowest and highest identity to SARS-CoV-2, respectively 162. The tertiary structure of the polyprotein isolate SARS-CoV-2_HKU-SZ-001_2020 had 98.94 % identity with SARS-Coronavirus NSP12 bound to NSP7 and NSP8 co-factors 163.

The analysis of fifteen sequences of SARS-CoV-2 S sequences obtained from NCBI and GISAID from China and various export locations worldwide along with representative members of lineages A-D betacoronaviruses showed that all SARS-CoV-2 S sequences clustered very closely with bat SARS-like sequences, with the closest matching sequence corresponding to Bat-SL-RaTG13 162. Pairwise comparison between SARS-CoV-2 S protein and that of BatCoV-RaTG13 and representative sequences from Guangxi pangolin (2017, abbreviated GX here) and Guangdong pangolin (2019, abbreviated GD) confirm that overall BatCoV-RaTG13 had the highest identity: 97% overall, 96% and 100% for S1 and S2, respectively 162. The analysis revealed that pangolin S protein sequences are more divergent overall (92% identity for GX and 89% identity for GD), with most of the divergence concentrating in the S1 domain. the RBD domain of the GD domain was confirmed to be remarkably well conserved compared to SARS-CoV-2 (97% identity compared to 87% identity for GX pangolin and 89% for BatCoV-RaTG13) 162. Another study showed that he homology of SARS-CoV-2 with the Bat coronavirus isolate RaTG13 strain (MN996532) was 96%, but has no more than 80% homology with other isolates of bat SARS‐like coronavirus 164.

Zhang et al. showed that some pangolin CoV genes show higher amino acid sequence identity to SARS-CoV-2 than to RaTG13 genes 39. The RBD region within the S1 which is conserved between Pangolin CoV and SARS-CoV2, is phylogenetically closer to pangolin-CoV than RaTG13 pointing potential similarity in their pathogenic properties 39. At the whole genome sequence level, pangolin CoV and SARSr-CoV RaTG13 show 91.02% and 96.2% similarity with SARS-CoV-2 but the S1 subunit of spike protein of pangolin CoV is more closely related to SARS-CoV-2 than SARSr-CoV RaTG13. Other studies have shown that the homology with a coronavirus strain isolated from pangolin was 99%, suggesting that SARS‐CoV‐2 might have originated from bat and pangolin might have served as the intermediate host between bat and human 165. The genetic analysis of the currently circulating strains of the pandemic have shown 99.98-100% similarity in their genomes, implying a recent shift to humans 161. Description of the epitopes distribution over the viral population might provide valuable information driving future researches aimed at setting efficient prophylactic strategies and/or the design of tool capable of differential diagnosis on the basis of serological tests.

Sequence alignment analysis provides evidence of high sequence homology for some of the investigated proteins. In addition, homology modelling of structural epitope mapping revealed a potential immunogenic value for specific sequences scoring a lower identity with SARS-CoV-2 nucleocapsid proteins 154. Accumulated evidence provide a molecular structural rationale for a potential role in conferring protection from SARS-CoV-2 infection and identifying potential candidates for the development of diagnostic tools. Further experimental studies are desired for a confidential evaluation of the postulated hypotheses. Finally, further studies employing purified forms of the spike proteins and/or its epitopes are needed and should be evaluated carefully.

## Current diagnostic tools for COVID-19

Currently, severeral diagnostic tests for coronavirus include RT-PCR, real-time reverse transcription PCR (rRT-PCR), reverse transcription loop-mediated isothermal amplification, as well as real-time RT-LAMP are used 166, 167. RT-LAMP was used to detect MERS-CoV, it is highly specific and has similar sensitivity to rRT-PCR 168,169.

The China National Health Commission, laboratory examinations established that nasopharyngeal and oropharyngeal swab tests, have become a standard assessment for diagnosis of COVID-19 infection 170. Two one-step quantitative RT-PCR (qRT-PCR) assays were developed to detect two different regions (ORF1b and N) of the SARS-CoV-2 genome, allowing for earlier identification of patients 170. Later, three novel RT-PCR assays targeting the RNA-dependent RNA polymerase (RdRp)/helicase (Hel), spike (S), and nucleocapsid (N) genes of SARS-CoV-2 were developed. Among them, the COVID-19-RdRp/Hel assay had the lowest limit of detection *in vitro* 171. The SARS-CoV E gene assay was more sensitive than the RdRp gene assay combined with the one-step RT-PCR system 172. The E gene PCR was sufficient to diagnose a SARS-CoV-2 infection but the RdRp protocol was recommended to confirm a positive result 173,174. The overall positive rate of RT-PCR detection of SARS-CoV-2 infection in 4880 cases from one hospital in Wuhan was 38% 175. In a series of 51 patients with confirmed COVID-19 infection, 71% patients were RT-PCR positive at the first time of testing of throat swab or sputum samples 176. The RT-PCR results usually become positive after several days (2-8 days) 177. Chest CT scans can be used to assess the severity of COVID- 19. Despite negative RT-PCR results, COVID-19 infection should be diagnosed with typical chest computerized tomography (CT) characteristics for patients suffering from fever, sore throat, fatigue, coughing or dyspnea 178. Of 1014 patients, 59% had positive RT-PCR results, and 88% had positive chest CT scans 179. Assessment of imaging features combined with clinical and laboratory findings could facilitate early diagnosis of COVID-19 pneumonia 180,181,182. The detection of nucleic acid in the nasal and throat swab sampling or other respiratory tract samplings by real-time PCR and further confirmed by next-generation sequencing is qualified as the most qualified method to diagnosis of COVID-19. Some published sequences comparisons of SARS-CoV-2 with other coronaviruses are presented in **Table 1.**

## Potential therapeutics for COVID-19

Until the writing of this paper, there is no current evidence from randomized controlled trials (RCTs) to recommend any specific anti-SARS-CoV-2 treatment for COVID-19 infection. Antiviral drugs and systemic corticosteroid treatment commonly used for influenza virus including neuraminidase inhibitors, ganciclovir, acyclovir, and ribavirin, as well as methylprednisolone 183 are invalid for COVID-19. However, other viral drugs are found to treat cases of COVID-19 such as remdesivir which has been reported to successfully treat the first case of COVID-19 in the US 184. Based on the experience accumulated from the SARS and MERS outbreaks, lopinavir (LPV) is a potential treatment option for COVID- 19, LPV inhibits the protease activity of coronavirus *in vitro* and in animal studies 185. Ribavirin, a guanosine analogue used to treat several virus infections, showed promising results in a MERS-CoV rhesus macaque model 186 and in SARS-CoV-2 RNA-dependent RNA polymerase (RdRp) model 187. These features increase its potential as an antiviral against SARS-CoV-2 188. Nelfinavir was predicted to be a potential inhibitor of SARS-CoV-2 main protease 188. The other promising antiviral drugs include nitazoxanide, favipiravir, nafamostat, and so on 189.

Chloroquine is a repurposed drug with great potential to treat COVID-19. It is a widely-used antimalarial and autoimmune disease drug that has been reported to be a potential broad-spectrum antiviral drug 190, 191, 192, and it has been used to treat malaria for many years 193. The chloroquine antiviral proprieties are investigated, it can inhibit pH-dependent steps of the replication of several viruses 194, 195, it has immunomodulatory effects, suppressing the production/release of TNF-α and IL-6, works as a novel class of autophagy inhibitor 196, which may interfere with viral infection and replication. Several studies have found that chloroquine interfered with the glycosylation of cellular receptors of SARS-CoV 195 and functioned at both entry and at post-entry stages of the COVID-19 infection in Vero E6 cells 197. Chloroquine was effective in more than 100 COVID-19 patients in terms of reduction of exacerbation of pneumonia, duration of symptoms and delay of viral clearance, all in the absence of severe side effects 198. Therefore, it has been included in the recommendations for the prevention and treatment of COVID-19 pneumonia 198. Hydroxychloroquine was found to be more potent than chloroquine Using physiologically-based pharmacokinetic (PBPK) models in SARS-CoV-2-infected Vero cells 199. Taken together, both chloroquine and hydroxychloroquine have immunomodulatory effects and can suppress the immune response 200, and may be used in prophylaxis as well as curative treatment for individuals exposed to SARS-CoV-2 201. The optimal dosage of chloroquine for SARS-CoV-2 will need to be assessed in future trials 201, in addition to the age of the patient and the clinical presentation or stage of the disease 202. In a study of 41 COVID-19 patients, 21% received corticosteroids, which could suppress lung inflammation 203, in respect to administered dose depending on disease severity 203. However, the clinical outcomes of coronavirus and similar outbreaks do not support the use of corticosteroids. In a retrospective observational study, 309 MERS patients given corticosteroids were more likely to require mechanical ventilation, vasopressors, and renal replacement therapy 204. Overall, there are many reasons to prevent treatment of patients with COVID-19 infection with corticosteroids, and such treatment may be harmful 205. Alternatively, Chinese clinicians combined Chinese and Western medicine treatment including lopinavir/ritonavir, arbidol, and Shufeng Jiedu Capsule (SFJDC, a traditional Chinese medicine) and gained significant improvement in pneumonia associated symptoms in Shanghai Public Health Clinical Center, China 206.

The development of vaccines and therapeutic antibodies against COVID-19 has important implications. The majority of the vaccines being developed for coronaviruses target the spike glycoprotein. The cross-reactivity of anti-SARS-CoV antibodies with the COVID-19 spike protein was assessed because SARS-CoV- 2 and SARS-CoV show a relatively high identity of the receptor-binding domain (RBD). The SARS-CoV-specific human monoclonal antibody CR3022 binds potently with the COVID-19 RBD 207. However, other SARS-CoV RBD-directed antibodies 230, m396 and 80R cannot bind to the COVID-19 RBD. Thus, Wrapp et al. suggest that CR3022 may be a potential therapeutic candidate, alone or in combination with other neutralizing antibodies, for the prevention and treatment of COVID-19 infections 208. Monoclonal antibodies can only recognize a single antigen epitope, which limits their use in the treatment of COVID-19. Applying monoclonal antibodies for new pathogens to clinical practice requires time. Alternatively, convalescent plasma has been suggested to treat COVID-19, the method was used early after symptom onset in the treatment of SARS, and the pooled odds of mortality following treatment was reduced compared with placebo or no therapy (odds ratio, 0.25) 209. Now, the structure of SARS-CoV-2 is revealed allowing to the development of medical countermeasures and optimization vaccination strategies.

## Future directions

The emerging pneumonia, COVID-19, caused by SARS-CoV-2, is a contagious pandemic. At the time of writing, the pandemic is still ongoing, with no available treatment. To battle this pandemic and be prepared for future outbreaks, it is crucial to understand its pathogenesis. SARS-CoV-2 exhibits strong infectivity but reduced virulence compared to SARS and MERS-CoVs. Fortunately, the scientific community has made progress in the characterization of the novel coronavirus and is working extensively on therapies and vaccines. Phylogenetic analyses suggest a bat origin of 2019-nCoV, but, considering the widespread distribution of SARS-CoVs in natural reservoirs, such as bats, camels, and pangolins, further research is needed to find novel intermediate hosts and block interspecies transmission. The genomic structure of the newly emerged coronavirus is evolutionary and may be under selective pressure with several mutations and homologous recombination. 2019-nCoV also potentially recognizes ACE2 from a diversity of animal species, implicating these animal species as possible intermediate hosts or animal models for 2019-nCoV infections. These results provide insights into the receptor usage, cell entry, host cell infectivity, and animal origin of 2019-nCoV. Furthermore, epidemic surveillance and preventive measures against SARS-CoV-2 will be enhanced. Structural studies on ACE2 receptor complexes with 2019-nCoV spike protein will contribute to understanding cross-species receptor usage of this novel coronavirus. *In vitro* studies will be useful for investigating how SARS-CoV-2 modifies gene expression in primary target cells, such as macrophages, dendritic cells, lymphocytes, and pulmonary epithelial cells. Animal models will allow investigators to determine the relationship between viral load and disease outcome, as well as to evaluate the role of infection and immune dysfunction in the disease process. Gained knowledge will facilitate the development of specific therapies designed to minimize pulmonary disease and optimize the anti-SARS-CoV-2 immune response. The striking structural similarity and sequence conservation among the SARS-CoV-2 S and SARS-CoV S glycoproteins emphasize the close relationship between these two viruses, which both recognize human ACE2 to enter target cells. This resemblance is further strengthened by the finding that SARS-CoV spike elicited polyclonal antibodies responses, potently neutralizing SARS-CoV-2 spike-mediated entry into cells 80. The SARS-CoV-2 RBD binds to ACE2 with a higher affinity than does the SARS-CoV RBD. Thus, potent SARS-CoV-specific neutralizing antibodies that target the receptor binding site of SARS-CoV failed to bind 2019-nCoV S protein, indicating that it is necessary to develop novel monoclonal antibodies that could bind specifically to the 2019-nCoV RBD 210. Other scientists indicate that the soluble ACE2 may act as a competitive interceptor of SARS-CoV and other coronaviruses by preventing binding of the viral particle to ACE2 211. Indeed, *in vitro* studies showed SARS-CoV replication was blocked by a soluble form of ACE2 in a monkey kidney cell line 212,213. Moreover, ACE2 fused to the Fc portion of immunoglobulin has just been reported to neutralize SARS-CoV-2 *in vitro* 214. In this context, soluble recombinant human ACE2 protein could actually be beneficial as a novel biologic therapeutic to combat or limit infection progression caused by coronaviruses that utilize ACE2 as a receptor.

## Figures and Tables

**Table 1:** Sequences similarities expressed as % identity, between SARS-CoV-2 and other coronaviruses

Other Coronaviruses	Bat	SARS	Pangolin	Camel	MERS-	Dromedarius	Bovine	H-Enteric	Canine	Avian
**Epitopes of SARS-CoV-2 nucleocapsid**										
KHWPQIAQ; FAPSASAFF	100	100	100	78.571	78.571	78.571	52.941	52.941	52.941	-
AQFAPSA; SAFFGMSR	100	100	71.429	78.571	78.571	78.571	52.941	52.941	52.941	-
AQFAPSA; SAFFGMSR	100	100	100	71.429	71.429	71.429	63.636	63.636	63.636	58.333
PKGFYAEG; SRGGSQASSR	100	100	100	61.111	61.111	61.111	61.111	48	48	100
QFAPSASAF; FGMSRIGM	100	100	100	81.818	81.818	81.818	50	50	50	53.846
QLPQGTTLPKGF; YAEGSRGGSQ	100	100	100	61.111	61.111	61.111	66.667	66.667	66.667	100
YNVTQAFGR; RGPEQTQGNF	100	100	100	63.158	63.158	63.158	58.824	58.824	58.824	-
**SARS CoV-2 spike protein**										
424-437	-	-	-	-	-	-	80	80	80	-
447-458	-	-	-	-	-	-	75	-	75	-
754-764	-	-	-	-	-	-	83.33	83.33	83.33	-
789-799	-	-	-	-	-	-	57.14	57.14	57.14	-
1139-1152	-	-	-	-	-	-	70	100	70	-
**SARS CoV-2 Spike protein (GI QHR63290)**										
AGO98871	-	-	-	-	-	-	38.42	-	-	-
QAY30020	-	-	-	-	-	-	-	-	37.68	-
ACJ35486	-	-	-	-	-	-	-	37.68	-	-
ACT10865	-	-	-	-	-	-	31.23	-	-	-
SARS-CoV-2_HKU-SZ-001_2020	-	98.94	-	-	-	-	-	-	-	-
SARS-CoV-2 strain	-	-	99	-	-	-	-	-	-	-
**SARS-CoV-2 (Wuhan-Hu-1)**										
ORF1ab	95	86	-	-	50	-	-	-	-	-
S	80	76	-	-	30	-	-	-	-	-
ORF3a	91	72	-	-	-	-	-	-	-	-
E	100	94	-	-	36	-	-	-	-	-
M	98	90	-	-	42	-	-	-	-	-
ORF6	93	68	-	-	-	-	-	-	-	-
ORF7a	88	85	-	-	-	-	-	-	-	-
ORF8	94	40	-	-	-	-	-	-	-	-
N	94	90	-	-	48	-	-	-	-	-
